# Microbial signature of pediatric Crohn's disease: Differentiation from functional gastrointestinal disorders and relationship with increased disease activity

**DOI:** 10.14814/phy2.70665

**Published:** 2026-01-02

**Authors:** Jeremiah Levine, Scott C. Thomas, Fangxi Xu, Adam Isbiroglu, Ryan Zanganeh, Lauren Barazani, Mridula Vardhan, Samantha Hwang, Julia Kishanie Persaud, Nirali Thakor, Shelly Joseph, Leonardo Trasande, Deepak Saxena

**Affiliations:** ^1^ Division of Pediatric Gastroenterology NYU Grossman School of Medicine New York New York USA; ^2^ Department of Pediatrics NYU Grossman School of Medicine New York New York USA; ^3^ Department of Molecular Pathobiology NYU Dentistry New York New York USA; ^4^ Department of Environmental Medicine NYU Grossman School of Medicine New York New York USA; ^5^ NYU Wagner School of Public Service New York New York USA; ^6^ NYU College of Global Public Health New York New York USA; ^7^ Department of Surgery New York University Grossman School of Medicine New York New York USA; ^8^ Perlmutter Cancer Institute New York University Langone Medical Center New York New York USA

**Keywords:** Crohn's disease, microbiome, Pediatric Crohn's Disease Activity Index

## Abstract

The prevalence and incidence of Crohn's disease (CD) in pediatric populations have been steadily increasing. Growing evidence suggests that gut microbiomal community differences play a critical role in the pathogenesis of CD. Additionally, the clinical course of patients with CD is unpredictable, making treatment decisions challenging. We investigated the fecal microbiome of newly diagnosed, treatment‐naïve pediatric CD patients (*n* = 43) compared to age‐ and sex‐matched controls with other functional gastrointestinal disorders (*n* = 139). We also correlated microbial changes with CD disease activity, measured by the Pediatric Crohn's Disease Activity Index (PCDAI). Our results showed that microbial richness and diversity were significantly lower in CD patients. Furthermore, taxonomic analysis revealed an enrichment in pro‐inflammatory bacteria (*Fusobacteria* and *Proteobacteria*) and depletion in favorable bacteria (*Firmicutes* and *Verrucomicrobia*). Higher PCDAI scores were linked to the enrichment of genera harboring pro‐inflammatory taxa (*Hungatella* and *Veillonella*) and decreased abundance of genera harboring protective taxa (*Lachnospiraceae*). Our study underscores the potential of fecal microbiome profiling as an effective tool for understanding CD pathogenesis, identifying microbial biomarkers, and predicting disease activity for treatment response. This, in turn, can help to improve personalized treatment and management strategies in pediatric CD.

## 1 INTRODUCTION

Crohn's disease (CD) constitutes a complex and chronic inflammatory disorder affecting the gastrointestinal tract, attributed to the interplay between dysregulated immune responses and the intestinal microflora (Chu et al., 2016). It has been demonstrated that the incidence and prevalence of CD are increasing (Ng et al., 2017). Of concern, the incidence of pediatric CD has significantly increased, a trend not similarly observed in patients with ulcerative colitis (Benchimol et al., 2009). In 2018, the incidence of pediatric CD in North America was estimated to be 13.9 per 100,000 (Sýkora et al., 2018), significantly higher than the estimated 7.2 per 100,000 reported in studies conducted between 2007 and 2011 (Eszter Müller et al., 2014). Overall it is estimated that there is a 75% increase in pediatric diagnosis in the last 2 decades. (Touma et al., 2023) Furthermore, special considerations need to be made in the pediatric population, given the tendency for a more severe phenotype in those with earlier onset disease and the unique lifelong burden these patients may experience, with potential negative effects on growth, psychological/emotional function, and body habitus (Zhuang et al., 2021).

This rising incidence in CD cannot be readily explained by a single generational change in the human genome. Likely, there is a multifactorial interplay involving impaired mucosal immune response and capability of the mucosa to prevent bacterial translocation, diet, and environmental exposures (Maukonen et al., 2015). More recently, the critical role of the microbiome in the development of intestinal microbial community differences has gained increasing attention; specifically, the enrichment of pro‐inflammatory bacteria, the depletion of beneficial bacteria, and reduced bacterial richness and diversity (McIlroy et al., 2018). Prior studies examining intestinal microbiome composition in pediatric patients with CD have shown enrichment of proinflammatory *Proteobacteria* and *Fusobacteriota*, variable relative abundance of *Firmicutes*, and depletion of beneficial *Bacteroidota* (Gevers et al., 2014).

The Pediatric Crohn's Disease Activity Index (PCDAI) is a tool to assess disease severity by incorporating subjective reporting of symptoms, the presence of extra‐intestinal manifestations, physical exam findings, and laboratory data (Grant et al., 2021). A PCDAI score is calculated based on these results. PCDAI scores range from 0 to 100; the higher the score, the more active the disease. A score above or equal to 30 indicates moderate to severe CD (Hyams et al., 2005). Limited data is available on the assessment of the gut microbiome relating to PCDAI scores specifically in pediatric patient populations. Kowalska‐Duplaga et al. was not able to demonstrate a correlation between alpha and beta diversity and most clinical parameters. However, they were able to demonstrate differences in diversities between eight children with low calprotectin levels versus those with higher levels. In addition, there was some microbiome change noted in five patients with PCDAI scores <10. However, no trends were observed with increasing disease severity (Kowalska‐Duplaga et al., 2019).

Functional disorders of the gastrointestinal tract are a heterogeneous group clinically. These disorders are believed to result from complex interactions among the gut–brain axis, motility disturbances, visceral hypersensitivity, and psychosocial factors and are all classified together as Disorders of brain–gut interaction (DGBI) (Benninga et al., 2016). Clinically, DGBIs such as irritable bowel syndrome (IBS) and functional dyspepsia, are prevalent conditions characterized by chronic or recurrent gastrointestinal symptoms without identifiable structural damage or biochemical abnormalities (Benninga et al., 2016; Drossman & Hasler, 2016). Notably, DGBIs often present with symptoms similar to those of CD, including abdominal pain, bloating, and altered bowel habits, which can complicate differential diagnosis (Colombel et al., 2019; Drossman & Hasler, 2016). Such symptomatic overlap emphasizes the importance of careful clinical assessment to distinguish between DGBIs and CD (Vasant & Ford, 2020). Furthermore, the high prevalence (up to 25%) of DGBIs in the pediatric population makes them a pertinent control group as they provide a relevant comparison for understanding microbial differences associated with CD (Aziz et al., 2018; Vasant & Ford, 2020).

Accurate diagnosis of CD requires invasive procedures with intestinal sampling as the gold standard for diagnosis, As such, alternative noninvasive approaches like fecal microbiome characterization may be especially useful in the evaluation of children with possible CD. Previous studies of fecal microbiome characterization often lack critical components: treatment‐naïve patients, diverse sample populations, and properly matched control groups. Furthermore, microbiome characterization has been limited, despite its potential to uncover biomarkers that could predict disease activity and may further be of use in predicting disease course and treatment response (Zheng et al., 2022).

Hence, the goals of this study were to compare the fecal microbiome of treatment‐naïve pediatric CD patients with that of a cross‐matched control group of treatment‐naïve patients diagnosed with DGBIs by characterizing the fecal microbiome at the level of the bacterial phylum and genus level among our two cohorts. We then analyzed the association between fecal diversity and richness and disease phenotype. To this end, we characterized the fecal microbiome for differences in the relative abundance of bacterial taxa, revealing significant differences among our two cohorts. Lastly, we demonstrated a correlation between decreasing fecal microbial alpha diversity measures with increasing disease phenotype.

## 2 MATERIALS AND METHODS

### 2.1 Human subject recruitment and sample collections

The study was approved by the Institutional Review Board of the NYU Grossman School of Medicine. Consecutive patients were enrolled at NYU Langone's Laurence D. & Lori Weider Fink Children's Ambulatory Care Center or at any other NYU Langone Health facility or affiliates as they presented for diagnosis and treatment of any gastrointestinal‐related complaint. Providers asked families if they were interested in participating, and, if willing, were approached by the research coordinator and/or nurse to review the study, complete the questionnaires and obtain help in collecting the specimens. Patients or legal representatives of non‐adult patients provided written informed consent to participate in the study.

We then identified two groups in the present study: newly diagnosed patients with CD and newly diagnosed patients with functional abdominal disorders in which other pathology was ruled out clinically. No patient was on medications including acid suppression, prokinetics, and probiotics and none were on specific dietary therapy. The goal was to differentiate cases and controls most definitively and exclude other inflammatory gastrointestinal disorders or processes that could confound the identification of microbial differences between the two groups.

CD was diagnosed by established criteria, using a combination of family and clinical history, physical examination, and laboratory, imaging, and endoscopic assessment (Stange, 2006). If the patient also had no prior history of CD‐directed treatment, they were recruited as a CD case. Among the cohort of patients with CD, PCDAI was used to further characterize the severity of clinical disease. Our DGBI group consisted of newly diagnosed and treatment‐naïve patients who met diagnostic criteria for a functional gastrointestinal disorder according to Rome criteria (Lacy & Patel, 2017). All patients were reviewed by a single physician to ensure uniformity in diagnosis, and the control patients were assigned an H‐classification based on Rome criteria. All control patients were then rereviewed by a second independent physician and H‐classification was reassigned.

We frequency matched cases with controls by age, sex, race, ethnicity, and insurance status. Fecal samples were self‐collected at home by participants using the OMNIgene•GUT|OM‐200 kit (DNA Genotek; Cat. No. OM‐200). Samples were shipped to the lab of Dr. Deepak Saxena using a provided Clinical pak with overnight priority. Upon receipt, samples were immediately stored at −80°C until further processing.

### 2.2 Fecal sample DNA extraction and 16S rDNA sequencing

Microbial DNA from patient fecal samples was extracted using QIAmp PowerFecal DNA extraction kits (Qiagen, Hilden, Germany; Cat. No. 12830‐50) as per the manufacturer's instructions and stored at −20C. Extracted DNA was checked for purity by Nanodrop and fluorometrically quantified using Quant‐iT™ PicoGreen™ dsDNA Assay Kits (Invitrogen, Waltham, MA, USA; Cat. No. P7589). The V3‐V4 region of the 16S rRNA gene was targeted for amplification and subsequent sequencing using 341F (5′‐CCTACGGGNGGCWGCAG‐3′) and 805R (5′‐GACTACHVGGGTATCTAATCC‐3′) as described in the study by Pushalkar et al. (2018). Briefly, samples were normalized to 10 ng/μL of DNA and sequenced according to a modified Illumina 16S metagenomics protocol (Part # 15044223 Rev. B). Indexed samples were pooled in equimolar amounts and sequenced on an Illumina MiSeq platform using MiSeq Reagent Kit v3 (600 cycles) following the 2 × 300‐bp paired‐end sequencing protocol. Negative controls consisted of DNA elution buffer and were processed in tandem with samples for every sequencing run. Paired‐end raw sequencing reads were quality‐checked using FastQC and MultiQC and analyzed using QIIME2 v2021.11.0 (Bolyen et al., 2019). Dada2 was used to denoise reads and generate an amplicon sequence variant (ASV) table using qiime dada2 denoise‐paired command with the following parameters: –p‐trunc‐len‐f 285 –p‐trunc‐len‐r 234 –p‐trim‐left‐f 20–p‐trim‐left‐r 21. A phylogenetic tree was created with FastTree using MAFFT alignment with qiime phylogeny align‐to‐tree‐mafft‐fasttree. For taxonomic classification, a QIIME2‐compatible SILVA 16S rRNA gene database was prepared using RESCRIPt based on the SSU Ref NR 99 138 dataset. A 16S rRNA v3‐v4 region‐specific classifier for taxonomic classification was trained using qiime feature‐classifier fit‐classifier‐naive‐bayes command with the primers 341F 5′‐CCTACGGGNGGCWGCAG‐3′ and 806R 3′‐GACTACHVGGGTATCTAATCC‐5′ (Bolyen et al., 2019; McMurdie & Holmes, 2012). The amplicon‐region specific classifier was then used to classify the DADA2 representative sequences to profile taxonomy using qiime feature‐classifier classify‐sklearn command. The ASV count table, taxonomy mapping file, and nwk tree file were imported into a phyloseq object for downstream analysis in R (McMurdie & Holmes, 2012).

### 2.3 Statistical analysis

Alpha‐diversity analysis was performed based on the number of observed Amplicon Sequence Variants (ASVs), Shannon index, and Faith's phylogenetic diversity measurements (Faith, 1992). Differences across study groups were assessed by Wilcoxon rank sum test and Bonferroni adjusted *p* values were reported. Beta‐diversity analysis was performed based on both Weighted UniFrac distance and Bray–Curtis dissimilarity matrix and visualized using a principal coordinates analysis (PCoA). Statistical significance between clusters was determined by permutational multivariate analysis of variance (PERMANOVA) using the Adonis function in the vegan R package (v2.6‐8). Differentially relative abundant taxa between groups were assessed using the Wilcoxon rank sum test and Linear discriminant analysis Effect Size (LEfSe) using the MicrobiotaProcess R package (v1.12.4) (Segata et al., 2011; Strauss et al., 2011). Additionally, Spearman correlation analysis was conducted to assess the associations between PCDAI scores and alpha diversity indices as well as the relative abundance of bacterial genera. Significant correlations (*p* < 0.05) between PCDAI scores and bacterial genera were visualized using linear regression lines and depicted as networks in Cytoscape (v3.10.1).

## 3 RESULTS

Our subjects ranged in age from 5 to 20 years old, consisting of a cohort with CD (*n* = 43) and cross‐matched control subjects with a diagnosis of a DGBI (*n* = 139). Participant demographics are outlined in Table 1. The patients with newly diagnosed Crohn's disease had BMI's that were significantly decreased compared to those with newly diagnosed DGBI. There were no other significant differences between the CD and DGBI cohorts. Fecal samples were collected within 1–2 weeks of diagnosis but not at the time of diagnostic endoscopy. A total of 182 fecal samples were collected and sequenced.

**TABLE 1:** Demographics.

Variable	CD (*n* = 43)	DGBI (*n* = 139)	Comparison
Mean age (SD) at consent	11.977 (3.342)	11.957 (3.453)	*p* > 0.99
BMI percentile (SD) at consent	38.41 (34.31)	54.93 (30.66)	*p* = 0.0031
Female (%)	18 (41.9%)	58 (41.7%)	
Race (%)			*p* = 0.32
White	35 (81.4%)	102 (73.4%)	
Other	8 (18.6%)	37 (26.6%)	
Refused/Unknown	0 (0.0%)	0 (0.0%)	
Private insurance (%)	21 (48.8%)	73 (52.5%)	*p* = 0.73
Further characterizations	PCDAI Scores (*n* = 41^a^): Range = 5–60 Mean = 31 Standard deviation = 13.6 Median = 32.5 Mode = 35	H‐Classifications: H1B = 2 H1C = 1 H1D = 1 H2A = 35 H2B = 39 H2D = 35 H3A = 22 H3B = 4	

^a^ There is insufficient data to calculate PCDAI scores for two CD patients.

### 3.1 Fecal microbiome in CD vs. DGBI

#### 3.1.1 Alpha and Beta diversity

**FIGURE 1:**
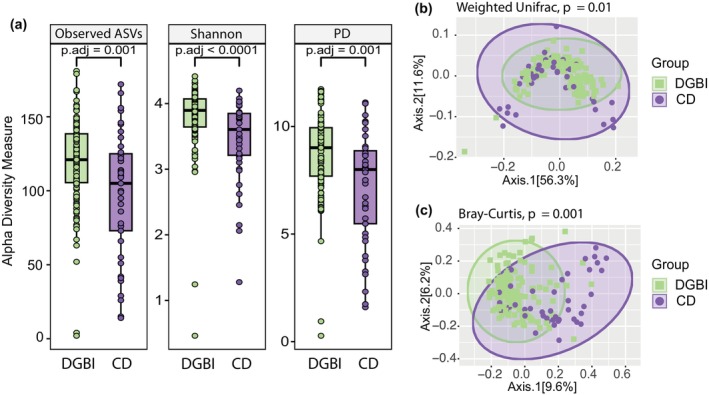
The fecal microbiome of patients with CD exhibits a reduction in alpha diversity measures compared to DBGI patients, and a significantly different microbial community structure. (a) Alpha diversity analysis at the species level (ASV) illustrating the differences measured by Observed ASVs, Shannon Diversity Index analysis, and Phylogenetic Diversity metric between CD and DGBI patients. Species‐level (ASV) beta diversity (Weighted Unifrac distance (b), Bray–Curtis dissimilarity (c)) principal coordinate plot, illustrating the difference in fecal microbiome community composition between CD and DGBI patients (PERMANOVA, *p* = 0.01).

Alpha diversity analysis of the fecal microbiome revealed significant differences in the richness and diversity between DGBI and CD cohorts. The CD cohort was significantly lower in all alpha diversity measures compared to the DGBI cohort (Shannon index (*p* < 0.0001), Faith's PD index (*p* < 0.01), and observed ASVs (*p* < 0.01)) (Figure 1a). Beta diversity, calculated using Weighted Unifrac distance and Bray–Curtis dissimilarity matrix and visualized via principal coordinates analysis (PCoA), revealed a significant difference in bacterial community composition between the cohorts (*p* = 0.01, *p* = 0.001, respectively) (Figure 1b,c).

#### 3.1.2 Bacterial composition

**FIGURE 2:**
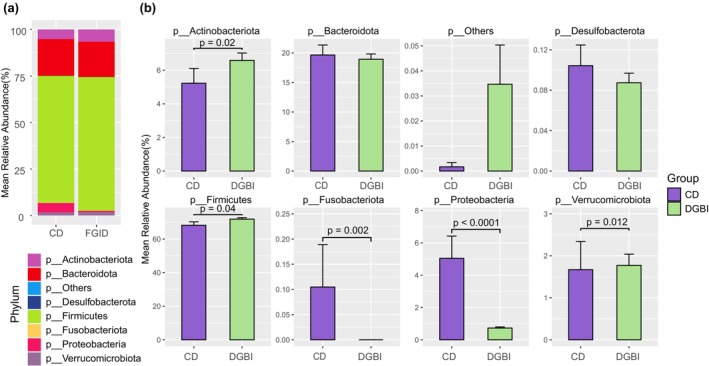
Phylum‐level analysis of fecal microbiome in patients with CD versus Functional GI Disorders. (a) Phylum‐level analysis illustrating the overall difference in the relative abundance of presented phyla between CD and DGBI patients. p_Others grouping, phyla with mean relative abundance less than 0.1% in all comparative groups (b) Mean relative abundances illustrating the significant differences amongst fecal microbial phyla between CD and DGBI cohorts.

The phylum‐level bacterial composition of CD and DGBI was examined, with the phyla *Firmicutes* and *Bacteroidota* comprising greater than 80% of the community (Figure 2a). Phylum‐level taxonomic differential relative abundance analysis revealed significant enrichment of phyla harboring pro‐inflammatory bacteria in our CD cohort (Figure 2b). These enriched phyla include *Fusobacteria* (*p* < 0.01) and *Proteobacteria* (*p* < 0.0001). In contrast, *Firmicutes* (*p* < 0.05), *Verrucomicrobia* (*p* < 0.05), and *Actinobacteria* (*p* < 0.05) are depleted in the CD cohort compared to DGBI.

**FIGURE 3:**
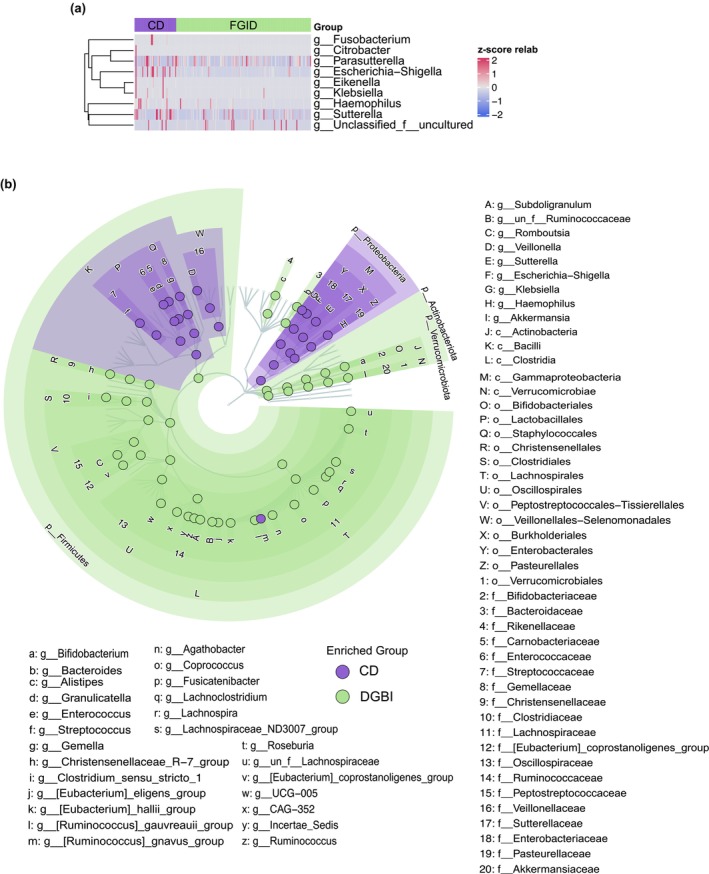
Differential abundance testing revealed significantly different taxonomic composition. (a) Individual sample‐based heatmap of all genera within *Fusobacteriota* and *Proteobacteria* between CD and DGBI cohorts. (b) LEfSe analysis‐generated cladogram highlighting the significantly enriched taxa between CD and DGBI cohorts.

Further, we describe significant differences in the genus‐level composition of the *Proteobacteria* and *Fusobacteria* phyla within both CD and DGBI (Figure 3a, Table S1). Among the *Proteobacteria* phylum, we demonstrate a significant enrichment of *Haemophilus* (*p* < 0.001), *Eikenella* (*p* < 0.00001), and *Klebsiella* (*p* < 0.001) in our CD cohort. *Escherichia‐Shigella* was found to have a nonsignificant difference in our fecal microbial profile. Within the *Fusobacteria* phylum, we note a significant enrichment in the CD cohort of the genus *Fusobacterium* (*p* < 0.05), which harbors many pathogenic species.

The LEfSe analysis in Figure 3b highlights significantly different taxonomic clades within the *Firmicutes* and *Proteobacteria* phyla, revealing retained phylogenetic differences at the genus level between cohorts. Within the *Proteobacteria* phylum, the genera *Klebsiella*, *Escherichia‐Shigella*, *Sutterella*, and *Hemophilus* are enriched in the CD cohort. Within the *Firmicutes* phylum, the genera *Veillonella*, *Gemella*, *Granulicatella*, *Enterococcus*, and *Streptococcus* are enriched in the CD cohort. These results further support our differential relative abundance testing above.

### 3.2 Increasing disease severity correlates with a decreased fecal microbiome diversity in CD

#### 3.2.1 Alpha diversity and PCDAI score

**FIGURE 4:**
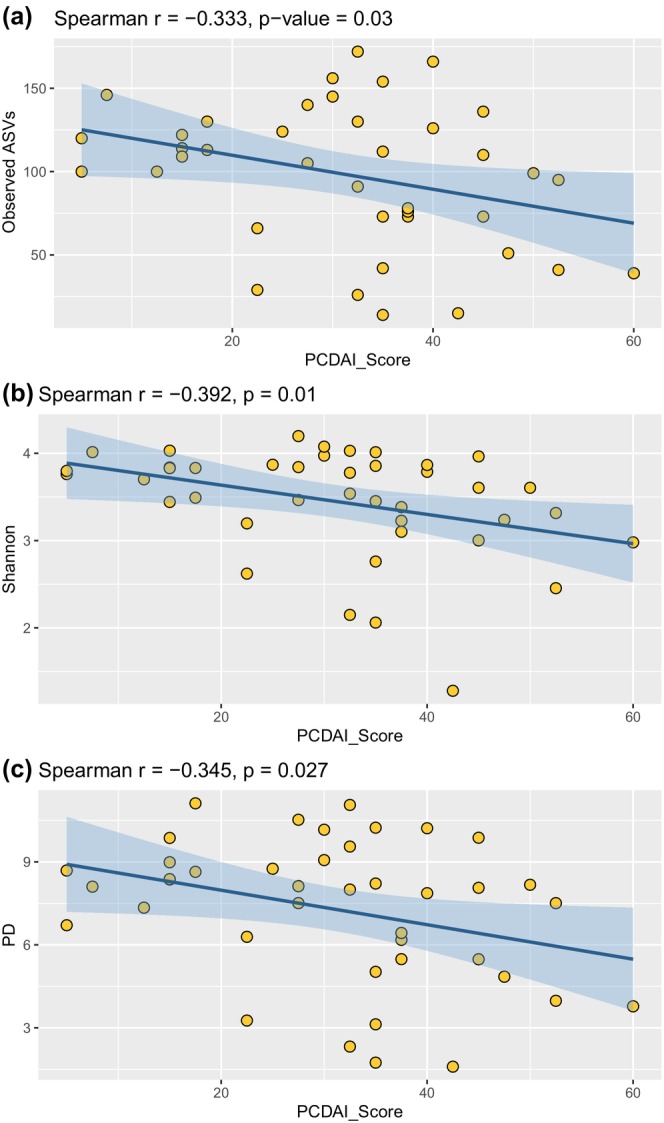
Spearman correlation analysis between disease severity PCDAI scores and fecal microbial alpha diversity in CD patients, as measured by (a) Observed Amplicon Sequence Variants (ASVs), (b) Shannon Diversity Index, and (c) Phylogenetic Diversity (PD). Each panel demonstrates a decrease in microbial diversity with increasing PCDAI scores (worsening disease severity).

We conducted a correlation analysis to assess the relationship between alpha diversity and disease severity, as measured by PCDAI scores. Our analysis demonstrates significant negative correlations between microbial alpha diversity measures and disease phenotype (Figure 4, Table S2). Significant negative correlations between disease phenotype and the Shannon diversity index (*p* < 0.01), observed ASVs (*p* < 0.001), and measures of PD (*p* < 0.01) were observed.

#### 3.2.2 Microbiota correlations with PCDAI score

**FIGURE 5:**
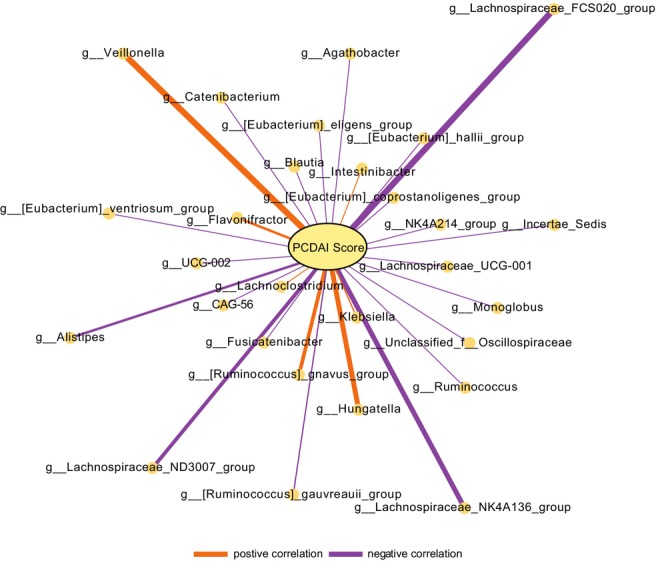
Spearman correlation analysis showing significant correlations (*p* < 0.05) between PCDAI score and specific bacterial genera in CD patients. The thickness of the vectors indicates the strength of the correlation (|correlation coefficient|). The greater the thickness of the vector, the greater strength of correlation. Purple vectors represent negative correlations, while orange vectors represent positive correlations.

Among CD cases, PCDAI scores were correlated to specific bacterial genera of the fecal microbiome (Figure 5). Notably, higher PCDAI scores were associated with increased relative abundance of genus *Veillonella* (*p* < 0.01) and *Hungatella* (*p* < 0.05), suggesting a potential role in disease activity. In contrast, higher PCDAI scores were associated with decreased relative abundance of *Lachnospiraceae*, most significantly the FCS020 (*p* < 0.001) and NK4A136 (*p* < 0.01) groups.

## 4 DISCUSSION

Our case–control study provides key insights regarding the fecal microbiome composition between treatment‐naïve pediatric patients with CD compared to patients with DGBIs, highlighting significant differences in microbial richness and diversity. Shannon diversity index, observed ASVs, and phylogenic diversity measures all demonstrated that reduced alpha diversity was significantly associated with worsening clinical phenotypes.

Our findings align with previous studies of the pediatric intestinal microbiome in patients with CD. Specifically, the observed enrichment of *Proteobacteria* and *Fusobacteria*, containing pro‐inflammatory taxa, as well as the depletion of *Firmicutes* and *Verrucomicrobia* (Gevers et al., 2014; Zhuang et al., 2021). Notably, the enrichment of *Fusobacteria*, particularly species such as *F*. *nucleatum*, suggests a potential pathogenic role in exacerbating inflammation through mechanisms involving tissue adhesion and irritant production (Elmaghrawy et al., 2022). Future longitudinal studies may help identify taxa in the phylum *Fusobacteria* that may serve to be clinically useful as a potential biomarker for CD diagnosis and activity (Strauss et al., 2011; Zhuang et al., 2021).

Additionally, enrichment of the *Proteobacteria* taxa *Klebsiella* and *Haemophilus*, within the CD cohort, is consistent with their known pro‐inflammatory interactions in CD (Fitzgerald et al., 2021; Harrison et al., 2012; Mirsepasi‐Lauridsen et al., 2019; Segata et al., 2011). *Klebsiella* has previously been shown to be elevated in the oral microbiome of CD patients, and its role in the pathogenesis of IBD may be related to its induction of T helper 1 cells (Fitzgerald et al., 2021; Harrison et al., 2012). Conversely, we observed a nonsignificant difference in *Escherichia‐Shigella* relative abundance which raises questions about its utility as a biomarker in fecal samples. *Klebsiella*'s presence in both the oral and intestinal microbiomes suggests that extraintestinal microbial sources can influence intestinal microbial community structure (Fitzgerald et al., 2021; Segata et al., 2011). Hence, a greater understanding of this interrelationship is necessary to open new avenues for microbial intervention targets.

Furthermore, our results revealed correlations between specific bacterial genera and disease severity in CD. Elevated levels of both *Hungatella* and *Veillonella*, which harbor species capable of inducing an inflammatory response, correlated with higher PCDAI scores suggesting the potential for them to play a role in increasing inflammation and contributing to a more severe disease phenotype (Atarashi et al., 2017; Lloyd‐Price et al., 2019; Santiago et al., 2023). Concomitantly or alternatively, these taxa may have increased fitness under the conditions of an inflamed intestine. Conversely, we found a decreased abundance of genera in the family Lachnospiraceae, specifically the genus‐level groups FCS020 and NK4A136. This suggests that they may play a protective role against severe disease due to their anti‐inflammatory and mucosal barrier‐enhancing properties (Lloyd‐Price et al., 2019; Maukonen et al., 2015; Olbjørn et al., 2019; Segata et al., 2011). Thus, these genera could serve as specific targets for therapeutic interventions to regain homeostasis.

A previous study did not demonstrate a correlation between microbial community structure and most clinical parameters including all levels of PCDAI (Kowalska‐Duplaga et al., 2019). However, they did suggest that there may be some correlation by using fecal calprotectin as a measure of disease activity. In addition, they used a small 5‐patient subgroup who did not have active disease to suggest that there may be abnormalities in patients with active disease. Our study, using larger numbers of patients and analyzing microbial composition to the level of the bacterial phylum and genus, is the first to demonstrate changes in fecal microbial alpha diversity and specific bacterial genera with worsening disease activity as demonstrated by increasing PCDAI.

Clinically, the strong correlation between microbial alpha diversity and disease severity demonstrates the potential for fecal microbiome characterization to be used as a noninvasive diagnostic and prognostic tool. Many current diagnostic procedures for patients with CD rely heavily on invasive endoscopic and colonoscopic procedures, which pose significant difficulties for pediatric patients (Bharadwaj et al., 2018). Therefore, fecal microbiome analysis may serve as a useful additive modality in the evaluation of children with newly diagnosed CD allowing for improved risk stratification (Schupack et al., 2022).

However, it is equally important to consider the limitations of our study. The dynamic nature of the fecal microbiome emphasizes the need for longitudinal sampling in order to accurately establish causal relationships between microbiome changes and disease progression (Grieneisen et al., 2023). Our study of cross‐sectional design limits us to assume how the fecal microbiome changed over time with regards to disease progression. Additionally, a larger number of patients would allow for a more accurate assessment of the correlation between the fecal microbiome and disease activity. Our DGBI control group had heterogeneous clinical manifestations, but recent studies have suggested that they all have similar pathophysiologic causes. While we leveraged 16S rDNA sequencing for its high throughput, it is limited in its taxonomic resolution. Future studies should strive for whole community sequencing comparing data between fecal microbiome changes in response to therapeutic interventions in pediatric patients with CD to enhance its clinical applicability.

In conclusion, our study demonstrates significant differences in fecal microbiome composition between pediatric patients with CD compared to a DGBI control group. Notably, we found that a decrease in microbial alpha diversity was associated with worsening disease severity. The enrichment of genera harboring pro‐inflammatory taxa, such as *Veillonella* and *Hungatella*, as well as the depletion of genera harboring protective taxa, such as *Lachnospiraceae*, provides a greater understanding of the microbiomes' role in CD pathogenesis and progression and warrant further mechanistic investigation. These results suggest that the fecal microbiome could serve as a biomarker for disease prognosis, severity, and treatment response for pediatric patients with CD. Our findings support the potential for fecal microbiome analysis to be used alongside current diagnostic tools in CD diagnosis and/or management in future hopes of using specific microbiome‐targeted therapies for pediatric patients with CD to improve their clinical outcomes.

## AUTHOR CONTRIBUTIONS

Jeremiah Levine, Leonardo Trasande, and Deepak Saxena conceived and designed research. Jeremiah Levine, Scott C. Thomas, Fangxi Xu, Samantha Hwang, Ryan Zanganeh, Shelly Joseph, Leonardo Trasande, and Deepak Saxena performed experiments. Jeremiah Levine, Scott C. Thomas, Fangxi Xu, Samantha Hwang, Ryan Zanganeh, Mridula Vardhan, Julia Kishanie Persaud, Nirali Thakor, Leonardo Trasande, and Deepak Saxena analyzed data and prepared figures. Jeremiah Levine, Scott C. Thomas, Fangxi Xu, Adam Isbiroglu, Ryan Zanganeh, Lauren Barazani, Leonardo Trasande, and Deepak Saxena drafted and revised the manuscript.

## FUNDING INFORMATION

This study was funded by The Leona M. and Harry B. Helmsley Charitable Trust (grant # 1911‐03329).

## CONFLICT OF INTEREST STATEMENT

None.

## ETHICS STATEMENT

This study was conducted in accordance with the ethical standards of New York University. Ethical approval for this research was obtained from the NYU Langone Institutional Review Board (IRB)/Ethics Committee of New York University under protocol number i19‐01024. All participants provided informed consent prior to their inclusion in the study. Participation was voluntary, and participants were informed that they could withdraw at any time without penalty.

## Supporting information


Table S1.

Table S2.

## Data Availability

Patient data are not publicly available due to HIPAA restrictions but are available to verified researchers upon request by contacting the corresponding author. The raw 16S rRNA gene sequencing data generated and analyzed in this study is available in the NCBI Sequence Read Archive (SRA) under BioProject accession number PRJNA1362478.
