# Supplementary material for: Microbial signature of pediatric Crohn's disease: Differentiation from functional gastrointestinal disorders and relationship with increased disease activity
Source: Physiol Rep. 2026 Jan 2;14(1):e70665. doi: 10.14814/phy2.70665 (PMC12759043; doi:10.14814/phy2.70665)
Supplement: Supplementary file 1 — Table S1. [file PHY2-14-e70665-s002.pdf]

Table S1. Significant genera within selected phyla (Fusobacteriota and Proteobacteria)

Genus	.y.	group1	group2	n1	n2	statistic	p	p.signif	p.adj	p.adj.signif	method
g__Eikenella	value	CD	FGID	43	139	3405.5	0.0000082	****	0.0000738	****	wilcox_test
g__Escherichia-Shigella	value	CD	FGID	43	139	3577	0.0434	*	0.1736	ns	wilcox_test
g__Fusobacterium	value	CD	FGID	43	139	3197	0.00179	**	0.01074	*	wilcox_test
g__Haemophilus	value	CD	FGID	43	139	3966	0.000091	****	0.000728	***	wilcox_test
g__Klebsiella	value	CD	FGID	43	139	3640	0.000102	***	0.000728	***	wilcox_test
g__Sutterella	value	CD	FGID	43	139	3546	0.0313	*	0.1565	ns	wilcox_test
