# Supplementary material for: Microbial signature of pediatric Crohn's disease: Differentiation from functional gastrointestinal disorders and relationship with increased disease activity
Source: Physiol Rep. 2026 Jan 2;14(1):e70665. doi: 10.14814/phy2.70665 (PMC12759043; doi:10.14814/phy2.70665)
Supplement: Supplementary file 2 — Table S2. [file PHY2-14-e70665-s001.pdf]

Table S2: Significant Spearman correlation between PCDAI score and bacterial genera

| row | column | cor | p |
|---|---|---|---|
| PCDAI Score | g__[Eubacterium]_coprostanoligenes_group | -0.336364818 | 0.031533707 |
| PCDAI Score | g__[Eubacterium]_eligens_group | -0.379596432 | 0.014371836 |
| PCDAI Score | g__[Eubacterium]_hallii_group | -0.390748528 | 0.011538483 |
| PCDAI Score | g__[Eubacterium]_ventriosum_group | -0.429247143 | 0.005101909 |
| PCDAI Score | g__[Ruminococcus]_gauvreauii_group | -0.434675513 | 0.004512728 |
| PCDAI Score | g__[Ruminococcus]_gnavus_group | 0.362777819 | 0.019747378 |
| PCDAI Score | g__Agathobacter | -0.420156827 | 0.006238574 |
| PCDAI Score | g__Alistipes | -0.453004432 | 0.002937858 |
| PCDAI Score | g__Blautia | -0.33563546 | 0.031927401 |
| PCDAI Score | g__CAG-56 | -0.361095867 | 0.020367381 |
| PCDAI Score | g__Catenibacterium | -0.41423585 | 0.00709144 |
| PCDAI Score | g__Flavonifractor | 0.337879029 | 0.03072913 |
| PCDAI Score | g__Fusicatenibacter | -0.354059379 | 0.023140894 |
| PCDAI Score | g__Hungatella | 0.375152996 | 0.015654582 |
| PCDAI Score | g__Incertae_Sedis | -0.430637143 | 0.004945015 |
| PCDAI Score | g__Intestinibacter | 0.309221586 | 0.049152747 |
| PCDAI Score | g__Klebsiella | 0.313363649 | 0.046040844 |
| PCDAI Score | g__Lachnoclostridium | 0.309223054 | 0.049151615 |
| PCDAI Score | g__Lachnospiraceae_FCS020_group | -0.503622023 | 0.000788832 |
| PCDAI Score | g__Lachnospiraceae_ND3007_group | -0.45423552 | 0.00285196 |
| PCDAI Score | g__Lachnospiraceae_NK4A136_group | -0.4596797 | 0.002497947 |
| PCDAI Score | g__Lachnospiraceae_UCG-001 | -0.358182307 | 0.021479975 |
| PCDAI Score | g__Monoglobus | -0.401614079 | 0.009250502 |
| PCDAI Score | g__NK4A214_group | -0.369105353 | 0.017554848 |
| PCDAI Score | g__Ruminococcus | -0.408063723 | 0.008085708 |
| PCDAI Score | g__UCG-002 | -0.346099063 | 0.026651411 |
| PCDAI Score | g__Unclassified_f__Oscillospiraceae | -0.323256155 | 0.039250481 |
| PCDAI Score | g__Veillonella | 0.466419175 | 0.002113601 |
